# Two Players in the Field: Hierarchical Model of Interaction between the Dopamine and Acetylcholine Signaling Systems in the Striatum

**DOI:** 10.3390/biomedicines9010025

**Published:** 2021-01-01

**Authors:** Jaromir Myslivecek

**Affiliations:** Institute of Physiology, First Faculty of Medicine, Charles University, Albertov 5, 12800 Prague, Czech Republic; jmys@lf1.cuni.cz; Tel.: +420-224-968-485

**Keywords:** dopamine receptors, muscarinic receptors, striatum, locomotor activity, addiction, biological rhythm

## Abstract

Tight interactions exist between dopamine and acetylcholine signaling in the striatum. Dopaminergic neurons express muscarinic and nicotinic receptors, and cholinergic interneurons express dopamine receptors. All neurons in the striatum are pacemakers. An increase in dopamine release is activated by stopping acetylcholine release. The coordinated timing or synchrony of the direct and indirect pathways is critical for refined movements. Changes in neurotransmitter ratios are considered a prominent factor in Parkinson’s disease. In general, drugs increase striatal dopamine release, and others can potentiate both dopamine and acetylcholine release. Both neurotransmitters and their receptors show diurnal variations. Recently, it was observed that reward function is modulated by the circadian system, and behavioral changes (hyperactivity and hypoactivity during the light and dark phases, respectively) are present in an animal model of Parkinson’s disease. The striatum is one of the key structures responsible for increased locomotion in the active (dark) period in mice lacking M_4_ muscarinic receptors. Thus, we propose here a hierarchical model of the interaction between dopamine and acetylcholine signaling systems in the striatum. The basis of this model is their functional morphology. The next highest mode of interaction between these two neurotransmitter systems is their interaction at the neurotransmitter/receptor/signaling level. Furthermore, these interactions contribute to locomotor activity regulation and reward behavior, and the topmost level of interaction represents their biological rhythmicity.

## 1. Introduction

The striatum is a structure belonging to the corticobasal ganglia [1] that has multiple functions. Some of these functions have been identified, and some of them have been partially discovered. The striatum plays roles in locomotor activity [2], addiction [3], memory [4], prediction and control of rewards, promotion of active defensive behavior [5], and sensory processing [6]; moreover, the caudate volume has been shown to have a significant positive correlation with intelligence [7].

From the functional morphological perspective, the striatum can be divided into the ventral and dorsal striatum. The ventral striatum consists of the nucleus accumbens and the olfactory tubercle. The dorsal striatum consists of the caudate nucleus and the putamen. The striatum can be further subdivided into sensorimotor and limbic/prefrontal (motivational) territories [8]. According to [9], three territories (or projection areas) can be found in the striatum: the associative, limbic, and motor territories.

Changes in neurotransmitter ratios (i.e., dopamine/acetylcholine [10] or dopamine/acetylcholine/glutamate) are considered prominent factors in striatal contributions to Parkinson’s disease [11], Huntington’s disease [12] and schizophrenia [1]. The level of acetylcholine in the striatum is 1 ± 0.5 nmol/L in F3×B6D2 mice [13], it should be taken into account that in the absence of brain acetylcholinesterase the level rises to 281 ± 167 nmol/L [13], which practically represents the maximal level of acetylcholine in mice striatum. The acetylcholine concentrations in humans can be measured indirectly by displacing the nicotinic radioligand by physostigmine [14] which is about 19% in normal healthy subjects. Similarly, dopamine levels in humans cannot be measured directly. Striatal dopamine level in mice (C57Bl/6J) is between 34.0 and 49.8 pmol/mg tissue [15]. In rat, the basal striatal dopamine level is 0.90 ± 0.16 nmol/L [16]. For changes in neurotransmitter levels (dopamine, acetylcholine) and its effects on specific striatal functions/disease, see Section 3.

The ventral striatum was shown to be involved in addiction [3], similar to the dorsal striatum, in which motor information is integrated with reward information [17]. In addition, the striatum also plays a role in autism development [18].

In many cases, there is a functional interconnection between dopamine neurotransmission receptors and cholinergic neurotransmission, mainly that of muscarinic acetylcholine receptors [1,3,11,19,20,21,22,23,24,25]. This is obviously the result of morphological interconnections between dopamine neurons and cholinergic interneurons. Cholinergic interneurons comprise less than 2% of total striatal neurons, are tonically active, and, as they have widespread axonal projections, modulate the activities of striatal outputs from medium spiny neurons [26].

Therefore, we will focus in this review on those aspects of striatal physiology in which the role of dopamine is tightly connected to acetylcholine, where these neurotransmitter systems play similar or opposite roles, and where the level of dopamine transmission depends on the level of acetylcholine signaling or vice versa. The functional interactions are established by the fact that all neurons in the basal ganglia are pacemakers [27]. Thus, not only constant connection between neurons but also rhythmic neuronal activity are the key principles on which striatal function is constituted. These interactions form specific functions and are specifically changed in locomotor or reward-related diseases. In this context, we propose here a hierarchical model of the interactions between the dopamine and acetylcholine signaling systems in the striatum.

## 2. Functional Morphology of Striatal Connections

**Figure 1:**
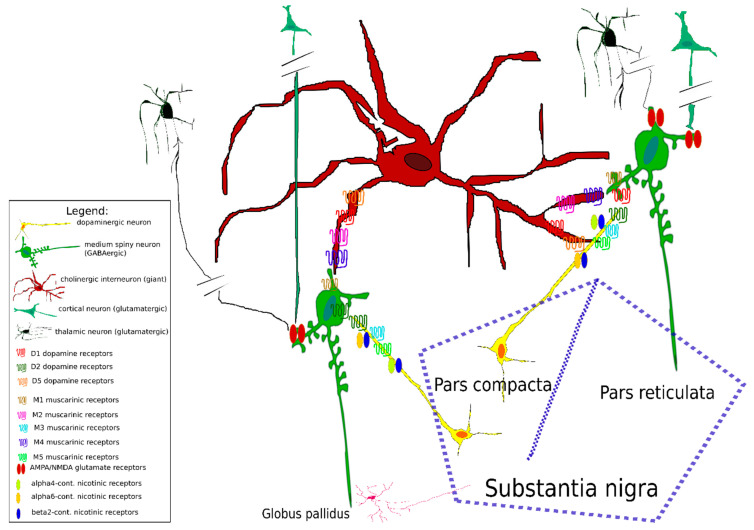
Schematic representation of interconnections between neurons in the striatum. The direct pathway projects preferentially to the globus pallidus internal segment (not shown)/substantia nigra pars reticulata, while the indirect pathway projects to the globus pallidus external segment. Efferentation to the entopeduncular nucleus is not shown. The substantia nigra (dotted blue line) is divided into the pars compacta and pars reticulata. The connections are simplified. Specific receptors are shown by symbols. Alpha4, alpha6, beta2-cont. nicotinic receptors: nicotinic receptors containing the α_4_, α_6_, β_2_ subunits, respectively. The neurons are schematized (see legend for explanation), and receptor localization is not specified (i.e., receptors are not shown to be expressed in nerve terminals or bodies). Please note that cholinergic neurons are also able to release glutamate. Both cholinergic and dopaminergic neurons can transmit the signal using volume transmission (see text); thus, the neurotransmitter can affect relatively remote receptors. Based on the data from [3,20,28,29,30,31,32,33,34].

The morphology of striatal connections is shown in Figure 1. In general, the main striatal neurons comprise dopaminergic medium spiny neurons that have specific efferentation (to pars reticulata, substantia nigra (the direct pathway) or to globus pallidus (the indirect pathway)) and cholinergic interneurons, which are important neuronal modulators of other (mainly medium spiny) neurons [3,20,28]. Furthermore, we will briefly describe the classic scheme of interaction between these neurons, and we will focus on some data that have cast doubt on this classic scheme. Then, we will describe the expression of specific receptors and signaling through receptors, and finally, we will mention some peculiarities in striatal neurotransmitter function that could affect dopamine-acetylcholine interactions in the striatum.

### 2.1. Classic Scheme of the Interaction between the Dopamine Pathway and Cholinergic Interneurons in the Striatum

The classic model suggests that activation of the “direct pathway” facilitates movement, and activation of the “indirect pathway” inhibits movement [35]. Some contradictory findings have cast doubt on the validity of this hypothesis [28]: the authors of these studies divided the medium spiny neurons into three types (see Table 1 and Figure 1) and described their almost ubiquitous efferentation to the substantia nigra, globus pallidus and entopeduncular nucleus. Other authors [27] described the existence of pacemaker cells in the striatum that exist as fast-spiking pacemakers (Na+ currents, hyperpolarization-activated cyclic nucleotide-gated (HCN) channels) or slow-spiking pacemakers (dopaminergic pacemakers, which are affected by cholinergic interneurons (also slow-spiking) and display two modes of pacemaking). They argued that the activity in the external and internal segments of the globus pallidus, the pars reticulata of the substantia nigra, and the subthalamic nucleus is autonomous. Striatal giant cholinergic interneurons are also autonomous pacemakers. The generation of a learning signal is thought to be caused by pauses in their activity. These pauses are generated by synaptically accelerated pacemaker activity in dopaminergic neurons of the substantia nigra pars compacta [27]. It also appears that oscillatory activity may play a significant role in the pathogenesis of movement diseases like Parkinson’s disease [36], and this activity can be reduced by dopaminergic treatments. However, a study of neural circuits of the basal ganglia [37] that used optogenetic control of direct and indirect pathways from medium spiny projection neurons revealed a more complex picture. Bilateral excitation of the indirect pathway elicited a parkinsonian state, and activation of the direct pathway reduced freezing and increased locomotion. These results and others described in the study [37] indicate that all responsive neurons in the pars reticulata of the substantia nigra showed robust changes in firing rate that were consistent with the classic model: direct pathway activation inhibited firing of substantia nigra and pars reticulata neurons, whereas indirect pathway activation excited substantia nigra and pars reticulata neurons.

Similarly, the classic scheme was complemented by the existence of rhythm-generating neurons, as reviewed by [10]. The authors discussed that normally, dopamine-dependent synaptic plasticity in striatal medium spiny neurons is made possible by a dramatic increase in dopamine release that is activated by the cessation of acetylcholine release. The synaptic plasticity of medium spiny neurons is lost after dopaminergic denervation. Another option is synchronized, rhythmic oscillatory activities in the nuclei of the specific circuits. Rhythmic firing of cholinergic interneurons and loss of acetylcholine release autoinhibition by M_4_ muscarinic receptors result in the unregulated release of acetylcholine. Thus, some authors concluded that the coordinated timing or synchrony of the direct and indirect pathways is critical for refined movements [35]. They also proposed a model in which the direct and indirect pathways act as differentiator circuits.

### 2.2. Expression of Specific Receptors and Signaling through Receptors

Acetylcholine release from cholinergic interneurons activates muscarinic receptors and has multiple effects that oppose dopamine release, signaling, and related motor behaviors [38]. This led to the formulation of the hypothesis that a balance between dopamine and acetylcholine signaling in the striatum is critical for maintaining normal motor function.

In a study that investigated the regulation of dopamine release from three types of midbrain neurons projecting to the striatum (dopaminergic, dopaminergic/glutamatergic, and glutamatergic neurons) [29], it was found that M_5_ muscarinic receptors potentiated dopamine and glutamate release only from the dopaminergic and dopaminergic/glutamatergic neurons. M_2_ and M_4_ muscarinic receptors decreased nicotinic-dependent dopamine release. The basal ganglia act on circuits; thus, for example, there is feedback control of the direct pathway from the substantia nigra [30]. Extrastriatal cholinergic projections from the pedunculopontine nucleus to the substantia nigra pars reticulata act on M_4_ muscarinic receptors to oppose cAMP-dependent dopamine D_1_ receptor signaling in presynaptic terminals of direct pathway striatal spiny projection neurons. This leads to tonic inhibition of transmission at direct pathway synapses and D_1_ dopamine receptor-mediated activation of motor activity. Medium spiny neurons express M_1_ muscarinic receptors that inhibit GABA release to cholinergic interneurons [39].

To make the picture more complex, it is necessary to mention that there are other acetylcholine receptors (i.e., nicotinic acetylcholine receptors, which contain the α_4_ subunit [33], in the dorsal striatum) and further functional interactions between other nicotinic α_6_ subunit-containing receptors and G protein-coupled receptors (such as adenosine A_2A_ [31]) that are involved in the control of dopamine release. Another nicotinic receptor subunit involved in dopamine release is the β_2_ subunit [40]. In mice with this subunit deleted, dopamine release was greatly diminished. Moreover, facilitation of dopamine release at low frequency and pulse number was found in wild-type but was absent in β_2_ knockout mice. However, chronic nicotine exposure caused upregulation of α_4_β_2_ subunit-containing nicotinic receptors but downregulation of α_6_β_2_ subunit-containing nicotinic receptors [32]. Dopamine release was changed only in response to chronic treatment with high nicotine doses.

Another aspect of striatal signaling complexity is the fact that cholinergic interneurons release not only acetylcholine but also glutamate [41]. Transporters of acetylcholine (vesicular acetylcholine transporters) and glutamate (the vesicular glutamate transporter VGLUT3) influence the storage of neurotransmitters in cholinergic interneurons. These individual neurotransmitters regulate striatal computation and distinct aspects of behavior.

### 2.3. Some Peculiarities in Striatal Neurotransmitter Function

In the traditional view of the central nervous system, neurons communicate via classic synapses. However, at the end of the last century, two types of synapses were discovered in the central nervous system: open synapses (providing “volume transmission”) and closed synapses (providing “wired transmission”). Importantly, the presence of “volume transmission” or “open” synapses has been confirmed for both dopamine [42] and acetylcholine [43] transmission in the striatum. This type of connection between neurons differs from classic synapses, which are also called “wired transmission” or “closed” synapses [44]. Thus, dopamine or acetylcholine can diffuse for long distances (generally between 100 nm and 1 mm) in the striatum, while classic synapses only allow neurotransmitters to spread 20–50 nm. This means that dopamine or acetylcholine can affect not only neurons in close proximity but also other neurons as far as 1 mm from the release point that bear specific receptors for these neurotransmitters.

Cholinergic neurons in the striatum express two vesicular transporters that can load either acetylcholine or glutamate into synaptic vesicles [45]. Therefore, cholinergic interneurons can release both neurotransmitters. In the abovementioned report [41], the authors determined the specific roles of acetylcholine release by selective elimination of the vesicular acetylcholine transporter from striatal cholinergic neurons and found no alteration in locomotor activity. While elimination of VGLUT3 induced hyperlocomotion, studies in mice that cannot release acetylcholine from cholinergic interneurons showed no alterations in their locomotor activity. Elimination of the vesicular acetylcholine transporter led to behavioral sensitization and cocaine preference similar to those of control mice. However, in these mice, there was an increased response to D_1_ and D_2_ agonists (i.e., decreased dopamine release was compensated by increased dopamine receptor responses).

In another report, [45], the authors described that elimination of vesicular acetylcholine transporters had only marginal consequences in striatum-related tasks and did not affect spontaneous locomotion, cocaine-induced hyperactivity, or reward properties. However, the dopaminergic sensitivity of medium spiny neurons and the behavioral outputs in response to direct dopaminergic agonists were enhanced. These observations indicate that spontaneous locomotor activity and the reward response to cocaine are mediated by glutamate and not by acetylcholine release.

In addition to functional changes, diseases in dopamine/acetylcholine neurotransmitter systems can be characterized by morphological differences. Thus, it is possible to find changes in striatal medium spiny neurons both in diseases connected with motor impairment (Parkinson’s disease) and addiction. In Parkinson’s disease, striatal medium spiny neurons lose dendritic spines, and in chronic cocaine exposure, aberrant striatal spine growth occurs [46].

## 3. Specific Functional Interconnection between Dopamine Receptors and Muscarinic Receptors

As mentioned above, there is a tight interconnection between dopamine and acetylcholine signaling in the striatum. In this section, we will discuss some aspects of these interconnections. There are important changes in neurotransmitter levels. Both the dopamine level and the acetylcholine level are changed in specific functions or diseases. Most importantly, the acetylcholine level in the striatum correlates with locomotor activity [47]. In parkinsonian rat (after medial forebrain bundle axotomy depleting dopamine in the nigrostriatal pathway), the striatal level of acetylcholine increased [48] from approximately 125 nmol/L to 175 nmol/L. This is in accordance with the measurement of the acetylcholine level in different Parkinson disease-linked genes knockout rats in which PINK1 KO rats had decreased acetylcholine levels at age 12 months compared to 4 months [49]. Similarly, as reviewed by [50], the acetylcholine level is increased in Parkinson’s disease and also in Huntington’s disease. The increased level of acetylcholine in nucleus accumbens can influence drug intake, as well as cessation of feeding [51]. Dopamine level is decreased in Parkinson’s disease [50] and increased after addictive substances treatment [52,53]. For example, in rats, amphetamine sensitization led to increase in the dopamine level to 1.74 ± 0.19 nmol/L (basal level is 0.9, see above) [16]. As the dopamine level in humans cannot be measured directly, there are only indirect data on dopamine synthesis capacity [54], or indirect measurement based on the competition of PET ligands with dopamine receptors [17]. There are sub-regional differences in dopamine synthesis capacity between anterior caudate nucleus and posterior putamen, posterior caudate nucleus and anterior putamen, anterior caudate nucleus and posterior putamen, and nucleus accumbens and posterior putamen [54]. In Parkinson’s disease patients, there was a reduced dopamine synthesis capacity in the posterior putamen (bilaterally) [55]. Dopamine level in the human ventral striatum increases by 10–15% after intravenous amphetamine, by 10–22.5% after oral amphetamine, by 0.5–27.5% after nicotine, and by 8–15% after alcohol, as referenced by [17]).

We will focus on the main functional interconnections (i.e., locomotor activity and reward/addiction), and we omit some functions that are not as prominent. Furthermore, we will describe the specific functional properties (i.e., the biological rhythms) of both neurotransmitter systems that were first discovered in the 1980s, but their full importance and specificity have only been revealed in recent years. Moreover, changes in the timing, i.e., rhythmicity, of these systems are also attributed to diseases, including defects in both locomotor function and reward function.

### 3.1. Locomotor Activity

In pioneering work [56], it was shown that the subcellular particle with which some dopamine is associated is similar to but distinguishable from that with which acetylcholine is associated. This work also referred to the localization of acetylcholine in subcellular structures of the CNS. The first reports about the identification of dopamine receptors and muscarinic receptors in striatal structures appeared in the 1970s [57,58].

As dopaminergic and cholinergic neurons are tightly interconnected, as discussed above (see also Table 1 and Figure 1), it is not surprising that dopamine receptor numbers are correlated with the numbers of muscarinic receptors. Concerning the genes encoding these receptors, the first study indicated the expression of three muscarinic receptor mRNAs in the striatum and only one (D_2_) dopamine receptor mRNA [59]. Later, neurons bearing the m1, m2, and m4 muscarinic receptor genes were identified as cholinergic, enkephalinergic and dopaminergic neurons [60].

More recently, M_4_ muscarinic receptors in D_1_ receptor-expressing striatal neurons were shown to decrease locomotor activity [61]. Another report indicated intercellular signaling interactions between M_4_ muscarinic receptors and D_1_ dopamine receptors [62]. Another aspect of locomotor activity regulation was shown [63] in which a lack of M_1_ muscarinic receptors led to elevated dopaminergic transmission in the striatum and increased locomotor activity. Thus, not only M_4_ muscarinic receptors but also M_1_ muscarinic receptors contribute to the regulation of locomotor activity. Interestingly, M_1_ muscarinic receptor knockout mice, as reported previously [63], also had an increased response to the stimulatory effects of amphetamine, suggesting a connection between muscarinic and dopamine receptors in addiction.

A study on mice lacking specific subtypes of muscarinic receptors (M_1_–M_5_) identified the M_4_, M_3_, and M_5_ muscarinic receptor subtypes to be involved in striatal dopamine release [64]. However, these subtypes differed in their effects on dopamine release, which was abolished by M_4_ muscarinic receptors (located on neuronal cell bodies), increased by M_3_ muscarinic receptors (located on nerve terminals), and reduced but not abolished by M_5_ muscarinic receptors (located on nerve terminals). Moreover, M_3_ and M_4_ muscarinic receptors mediated their effects via facilitation or inhibition, respectively, of striatal GABA release. Another aspect of the interaction between muscarinic and dopamine receptors is the comodulation of glutamate input to medium spiny striatopallidal neurons [65].

The fact that M_4_ muscarinic receptors affect motor behavior in addition to addiction behavior was proven [22]. This study showed that M_4_ muscarinic receptors are coexpressed with D_1_ dopamine receptors in the same neurons. Then, the authors generated mutant mice that lack M_4_ muscarinic receptors in D_1_ dopamine receptor-expressing cells. These animals revealed behavioral phenotypes that include enhanced locomotor activity and increased behavioral sensitization following treatment with psychostimulants (see Section 3.2). In addition to these behavioral changes, there was a lack of muscarinic inhibition of D_1_ dopamine receptor-mediated cAMP stimulation in the striatum and an increase in dopamine efflux in the nucleus accumbens.

Carbon-fiber microelectrode detection of dopamine in mouse striatal slices [66] found that muscarinic agonists decreased dopamine release evoked by low-frequency stimuli but increased the sensitivity of dopamine release to presynaptic activity and even enhanced dopamine release evoked by high frequencies (via striatal nicotinic receptors on dopaminergic axons). M_2_ or M_4_ muscarinic receptors but not M_5_ muscarinic receptors in the caudate–putamen or M_4_ muscarinic receptors in the nucleus accumbens also contributed to the control of dopamine release. These data indicate that striatal muscarinic receptors, by inhibiting acetylcholine release from cholinergic interneurons, modify the activity of nicotinic acetylcholine receptors, which in turn leads to dopamine release.

Another aspect of the effects of M_5_ muscarinic receptors on dopaminergic neurons was also investigated [19]. In coronal slices of the striatum, potentiation of M_5_ muscarinic receptors with a positive allosteric modulator resulted in an inhibition of dopamine release; this result was opposite to that of somatodendritic M_5_ muscarinic receptor activation in the substantia nigra and pars compacta neurons, which led to increased neuronal firing.

Conversely, conditional knockout of D_2_ dopamine receptors decreased the number of striatal cholinergic interneurons and caused motor deficits [67].

However, dopamine transmission (release) is affected not only by striatal cholinergic interneurons and cortical glutamatergic neurons but also by forebrain acetylcholine, which regulates striatal dopamine release, and brainstem cholinergic inputs regulate the transition of dopamine neurons from the tonic firing mode to the burst firing mode [68]. Thus, dopamine transmission between other locomotor activities can trigger exploratory motor behavior. In mice lacking total forebrain acetylcholine, frequency-dependent striatal dopamine release was enhanced [68]. These mice were hyperactive in a novel environment, whereas mice lacking rostral brainstem acetylcholine were hypoactive. The removal of both cholinergic sources normalized exploratory motor behavior.

Experiments with whole-cell recordings in acutely dissociated neurons [69] showed that M_4_ muscarinic receptor activation increased the network activity of direct pathway neurons but not of indirect pathway neurons, as seen with calcium imaging techniques. In addition, there was an additive effect between the D_1_ dopamine receptors and M_4_ muscarinic receptors on the excitability of direct pathway neurons or, paradoxically, opposite effects that depended on the order of their activation.

In a study [25] that addressed the role of cholinergic interneuron activity in the expression of parkinsonian-like motor deficits, altered M_1_ and M_4_ muscarinic receptors (postsynaptic, expressed on direct medium spiny neurons (D_1_ dopamine receptor-bearing)) in the dorsal striatum was shown to play a central role in the appearance of motor symptoms in Parkinson’s disease.

Medium spiny neurons also express D_1_ dopamine receptors [23] and, together with M_4_ muscarinic receptors, affect cAMP signaling. M_4_ muscarinic receptors have a high efficacy on cAMP signaling and can shut down the positive cAMP response induced by dopamine. This supports the possibility that a pause in acetylcholine release is required for phasic dopamine signaling. This suggests that M_4_ muscarinic receptors could be a novel therapeutic target to treat hyperactivity disorders. Parkinson’s disease is usually treated with L-DOPA, which, with chronic administration, leads to L-DOPA-induced dyskinesia, which has an important effect on the cholinergic system. In one study [21] investigating the role of M_1_ and M_4_ muscarinic receptors in L-DOPA-induced dyskinesia, it was shown that both M_1_ and M_4_ muscarinic receptor antagonists reduced D_1_ dopamine receptor agonist-induced dyskinesia but not D_2_ dopamine receptor agonist-induced dyskinesia, suggesting that muscarinic blockade differentially affects medium spiny neuron firing in the absence of postsynaptic dopamine. In a report describing the cataleptic responses in M_4_ muscarinic receptor knockout mice, the authors observed a normal cataleptic response, but systemic administration of a muscarinic antagonist did not suppress the cataleptic response. These results suggest that acute, but not chronic, blockade of muscarinic M_4_ receptors plays important roles in the therapeutic effects of antimuscarinic agents. However, it is necessary to note that the muscarinic antagonist used in this study was not M_4_ muscarinic receptor-specific; thus, blockade of other muscarinic receptor subtypes may also have contributed to this effect. Consistent with the hypothesis about dopamine/acetylcholine balance [38], muscarinic receptor antagonists have efficacy in reducing motor symptoms in diseases where dopamine release or signaling is diminished (Parkinson’s disease, dystonia) but have adverse effects. As M_4_ muscarinic receptors play a central role in regulating dopamine signaling and release in the basal ganglia, M_4_ muscarinic receptor activity is a potent regulator of motor dysfunction [38]. The dopamine/acetylcholine balance hypothesis is now being revisited. As reviewed by [43], these neurotransmitters exhibit coincident changes in their activity. Furthermore, the cited review discussed that this concept is being revisited in light of the major role played by thalamostriatal connections in striatal microcircuits and synaptic rearrangements in Parkinson’s disease and the complexity of the receptors involved in these regulations. However, it is necessary to keep in mind that Parkinson’s disease is not a physiological condition; thus, the dopamine/acetylcholine balance hypothesis could still be valid, as discussed in [38,70,71,72].

### 3.2. Some Aspects of Addiction/Reward

Addiction is a complicated phenomenon that is not yet fully understood. Thus, we will focus on dopamine/acetylcholine (muscarinic/nicotinic) interconnections with respect to addiction only. A complete discussion of the addiction pathways in the striatum exceeds the scope of this review.

In general, drugs (stimulants, with the exception of cannabis and opiates) increase striatal dopamine release, which in turn decreases the numbers of dopamine receptors (D_2_ and D_3_) [17].

Some drugs can potentiate the electrically evoked release of both dopamine and acetylcholine [73]. Similarly, a balance between dopaminergic and cholinergic activity in the ventral striatum or nucleus accumbens appears to be important for optimal behavior [71], and acetylcholine in the nucleus accumbens may quench the effects of excessive dopamine release. Thus, the local, selective depletion of cholinergic interneurons caused a marked increase in the locomotor activating effects of amphetamine [71]. Another aspect of the interaction between the cholinergic and dopamine neurotransmitter systems has been shown by [74], who ablated cholinergic interneurons from the adult nucleus accumbens and examined the role of acetylcholine transmitters in adaptive behavioral changes associated with cocaine reinforcement and addiction. In mice lacking unilateral cholinergic cells, cocaine induced abnormal rotation that was enhanced by repeated exposure to cocaine. In mice lacking bilateral cholinergic cells, chronic cocaine administration induced a prominent and progressive increase in locomotor activity. In the same context, it was shown that catalepsy induced by antipsychotics was diminished (not abolished) when M_4_ muscarinic receptors were missing [75]. A muscarinic agonist further attenuated the cataleptic response in M_4_ knockout mice; thus, non-M_4_ muscarinic receptors participate in anti-cataleptic effects. In further research, it was shown that the allosteric enhancer of M_4_ muscarinic receptors inhibited cocaine self-administration and cocaine-induced striatal dopamine increases, which was abolished in M_4_ muscarinic receptor knockout mice and had no effect on motor performance [76]. A partial reduction in the allosteric enhancer effect was shown in dopamine D_1_ receptor-expressing neurons in M_4_ muscarinic receptor knockout mice. As mentioned above, M_4_ muscarinic receptors are coexpressed with D_1_ dopamine receptors in a specific subset of striatal projection neurons. Mutant mice with missing M_4_ muscarinic receptors in D_1_ dopamine receptor-expressing cells only displayed enhanced hyperlocomotor activity (see also Section 3.1) and increased behavioral sensitization following treatment with psychostimulants (amphetamine and cocaine) or decreased cataleptic responses to haloperidol and risperidone [22]. These results show common mechanisms between locomotor control and addiction involving M_4_ muscarinic receptors and D_1_ dopamine receptors. In mice lacking the M_5_ muscarinic receptor [24], decreased self-administration of low to moderate doses of cocaine was found.

Corticostriatal connections can also contribute to addiction mechanisms. It was shown [77] that withdrawal of mice from repeated amphetamine treatment caused chronic presynaptic depression in glutamate release. An amphetamine challenge reversed chronic presynaptic depression via dopamine D_1_ receptor-dependent paradoxical presynaptic potentiation, which led to increased corticostriatal activity in direct pathway medium spiny neurons. It is possible to conclude that a chronic decrease in corticostriatal activity during withdrawal is regulated by tonically active acetylcholine-releasing interneurons and returns to normal upon re-exposure to amphetamine, which may represent an additional source of drug motivation during abstinence. In mice lacking the M_1_ muscarinic receptor, the effects on dopaminergic transmission and locomotor behavior were investigated [63]. There was elevated dopaminergic transmission in the striatum and significantly increased locomotor activity. M_1_ muscarinic receptor-deficient mice also had an increased response to amphetamine.

Not only muscarinic receptors but also nicotinic receptors affect dopamine release (see above) and thus alter the ability of cocaine to reinforce behaviors. Moreover, cocaine has a low inhibitory effect on nicotinic receptors. This led to a study in which biologically relevant concentrations of cocaine mildly inhibited nicotinic receptor-mediated currents in dopaminergic neurons and consequently changed dopamine release in the dorsal and ventral striatum [78]. Similarly, local cholinergic interneuron projections to dopamine neuron axons in the striatum together with modulatory cholinergic input from the pedunculopontine and laterodorsal tegmentum to dopaminergic midbrain nuclei are involved in the effects of nicotine [79].

As mentioned above, drug abuse relates to the release of both dopamine and acetylcholine. Normally, the release of dopamine and acetylcholine work together to support motor learning and motivated behaviors. Imbalances in neurotransmitter release can contribute to drug addiction [70]. The effects of dopamine and acetylcholine can be either antagonistic or synergistic. Blockade of nicotinic receptors diminishes abnormal repetitive behaviors induced by psychomotor stimulants, and blockade of postsynaptic muscarinic acetylcholine receptors in the dorsomedial striatum exacerbates drug-induced stereotypy [70]. In vesicular acetylcholine transporter-overexpressing mice, drug-induced stereotypies were increased [70]. Thus, vesicular acetylcholine transporter-mediated increases in acetylcholine could be critical in exacerbating drug-induced stereotypic behaviors.

### 3.3. Biological Rhythm

Almost every cell in a living organism can form rhythmic activity [80]. Thus, the striatum is also able to produce rhythmic changes associated with different events (e.g., fast- and slow-spiking pacemakers, see above). These rhythmic changes can be described based on their rates. Striatal microcircuits potentially generate rhythms [81], and striatal projection neurons and fast-spiking interneurons are capable of generating oscillations. The level of dopaminergic tone determines the rates of oscillations [81]. As the striatum is one of the brain structures involved in locomotor control, it is possible to deduce that it would be involved in biological rhythms connected to motor activity, locomotion, or addiction (drug/reward seeking).

Structures involved in locomotor biological rhythm regulation have been gradually discovered. The main circadian pacemaker is localized in the hypothalamic suprachiasmatic nuclei (SCN) [82]. In addition, other brain structures have recently been identified (for references, see [2]) as important in locomotor biological rhythm regulation: the subparaventricular zone (SPVZ), the intergeniculate leaflet (IGL), and the posterior hypothalamic area (PHA). Conversely, locomotor activity can also be considered a non-photic entraining signal of circadian rhythmicity (for references, see [2]).

Thus, diurnal variation in dopamine release has been described [83]. However, these data were obtained in vitro, and thus, one should be careful when interpreting them. In the morning (9:00–9:30), the spontaneous dopamine release rates were characterized by initially low values that gradually increased (approximately 3-fold) over 2.5 h. In the afternoon (15:00–15:30), the release gradually declined (approximately 2.5-fold). Other authors [84] have not found a diurnal rhythm of dopamine levels in the caudate nucleus. On the other hand, activation of dopamine receptors has been demonstrated to modulate the circadian effects of light on locomotor activity in mice [85] and to regulate the expression of clock genes in the striatum [86]. A direct relationship [87] between extracellular dopamine levels and the rhythm of expression of the clock protein PERIOD2 (PER2) in the dorsal striatum in rats was demonstrated. The authors [87] showed that the peak of the daily rhythm of extracellular dopamine in the dorsal striatum preceded the peak of PER2 by approximately 6 h, both peaks occurred in the dark (active) period, and the rhythm was circadian. Specific manipulations blocked the rhythm of striatal PER2, but none of them had any effect on the PER2 rhythm in the suprachiasmatic nucleus. Similarly, PER2 overexpression was shown to affect gene expression (which does not necessarily mean that receptor binding sites are changed) after repeated methamphetamine treatment of D_1_ and D_2_ dopamine receptors and dopamine transporters [53].

The biological rhythms of dopamine receptors were investigated in the early 1980s. The authors of two studies reported rhythmic changes in dopamine receptors [88] in a normal light/dark regime and in dopamine receptors and muscarinic receptors [89] in control and sleep-deprived rats. Dopamine receptors (in fact, D_2_-like dopamine receptors) were only investigated in the striatum in both of these reports, and the other receptor density changes reported in the paper describing the effect of sleep deprivation were measured in the forebrain. There was an ultradian rhythm in dopamine receptors. However, this group showed different results in [88,89]. In the first report, the rhythm was ultradian with two peaks (one in the dark period and the second in the light period). In the second report, the rhythm was again ultradian, but in control rats, there were two peaks of maximal density in the light (i.e., non-active) period. This rhythm was slightly changed by sleep deprivation. In subsequent work, it was shown that the rhythm is endogenous and varies seasonally [90]. The dopamine D_3_ receptor was shown to exhibit 24-h variations in the ventral striatum and is thought to influence motivation and motor functions [34]. The molecular components of the circadian clock acting as regulators of dopamine D_3_ receptors were described as retinoic acid-related orphan receptor α and orphan receptor REV-ERBα, which is activated by retinoic acid-related orphan receptor α [34]. As mentioned above, the striatum is connected with addiction/reward functions. Recently, it was observed that the reward function is modulated by the circadian system [91]. Specific reward-related regions/networks included the medial prefrontal cortex, ventral striatum, putamen, and default mode network. A very recent study [52] found that acute administration of cocaine triggered reprogramming of circadian gene expression in the striatum, which involved the activation of peroxisome protein activator receptor gamma, and this receptor was altered in mice with cell-specific ablation of the dopamine D_2_ receptor in striatal medium spiny neurons. Another recent study [92] showed behavioral changes (significant hyperactivity and hypoactivity during the light and dark phases, respectively, leading to a change in circadian timing) in an animal model of the preclinical stage of Parkinson’s disease.

Similar to dopamine, acetylcholine has also revealed diurnal variations. In the rat caudate nucleus, it peaked at 24:00 (i.e., in the dark period) in a normal 12 h light/12 h dark regime (8:00–20:00) [93]. The trough occurred at 18:00. Acetylcholine is released from nerve terminals in the dorsal striatum (limbic-prefrontal territory) via stimulation of NMDA receptors, and this release is modulated by μ-opioid receptors [94]. In these experiments, two time slots were used (2 (morning) or 8 (afternoon) hours after light onset, i.e., both in the light period) in either the presence or absence of an inhibitor of dopamine synthesis. Blockade of μ-opioid receptors enhanced the NMDA-evoked release of acetylcholine. These responses were more pronounced in the afternoon than in the morning. When dopamine synthesis was blocked, the NMDA-evoked release of acetylcholine was increased, with a similar amplitude in the morning and afternoon experiments. When dopamine transmission was suppressed, the enhancement of NMDA-evoked acetylcholine release by μ-opioid blockade was completely blocked in the morning and only partially blocked in the afternoon. The authors thus concluded that the μ-opioid-inhibitory regulation of acetylcholine release follows diurnal rhythms. While dopamine is required for this regulation in the morning and afternoon, an additional dopamine-independent process is present only in the afternoon. Interestingly, there is not only diurnal variation in acetylcholine release but also in the number of neurons. It was further demonstrated by this group [8] that the percentage of cholinergic interneurons containing μ-opioid receptors rose from 32% in the morning to 80% in the afternoon. Consequently, the μ-opioid receptor-mediated inhibition of acetylcholine release was higher in the afternoon. Moreover, enkephalin tissue content was the lowest in the afternoon. It is, therefore, possible to conclude that by acting on μ-opioid receptors present on cholinergic interneurons, endogenously released enkephalin reduced acetylcholine release. The consequences of altered dopamine transmission were again studied by this group [95], and it was shown that the expression of μ-opioid receptors in cholinergic interneurons as well as acetylcholine release by μ-opioid receptors was preserved after 6-hydroxydopamine treatment and downregulated after cocaine treatment. The expression of μ-opioid receptors in output neurons was downregulated in the 6-hydroxydopamine lesion and either preserved upon acute cocaine treatment or upregulated upon chronic cocaine treatment. These authors concluded that alteration in cholinergic transmission by μ-opioid receptors might play a major role in the motivational and cognitive disorders associated with dopamine dysfunction in fronto-cortico-basal ganglia circuits. Another paper reported [96] that vesicular glutamate transporter 3 knockout mice showed circadian-dependent hyperlocomotor activity that was restricted to the waking cycle and was due to an increase in striatal dopamine synthesis, packaging, and release. Vesicular glutamate transporter 3 (VGLUT3, see also above) is expressed by cholinergic interneurons in the striatum, where it regulates dopamine signaling. Conditional VGLUT3 knockout mice did not display changed dopamine release in the dorsal striatum or baseline locomotor activity, but they displayed changes in rearing behavior and sensorimotor gating. The elevation of dopamine release in the global knockout raised the possibility that motor deficits in a Parkinson’s disease model would be reduced by this knockout, which was confirmed, as in VGLUT3 KO mice (in contrast to WT mice), 6-OHDA-mediated dopamine depletion led to normal motor behavior across the entire circadian cycle. The roles of striatal cholinergic interneurons in generating beta and gamma oscillations in cortical-striatal circuits and in influencing movement behavior were investigated [97]. The authors found that selective stimulation of cholinergic interneurons amplified beta and gamma oscillations. Beta oscillations were supported through a muscarinic receptor-mediated mechanism. Activation of striatal cholinergic interneurons led to parkinsonian-like motor deficits in normal mice.

Muscarinic receptors have also been found to show circadian variation [98] in the striatum. Similarly, [99] found circadian rhythmicity and described that muscarinic receptor levels in the striatum were lower at 08:00 (light period) than at 22:00 (dark period). We identified the striatum as one of the key structures responsible for increased locomotion in the active (dark) period in mice lacking M_4_ muscarinic receptors [100]. The striatum was the only structure in which M_1_ muscarinic receptors played a role in locomotor biological rhythm regulation; in others (i.e., in the thalamus and intergeniculate leaflet), only M_4_ muscarinic receptors were responsible for this regulation. In our previous report [101], we did not find any difference between striatal D_1_-like and D_2_-like dopamine receptors measured at 9:00 AM and 9:00 PM, which, of course, does not necessarily mean that there is no biological rhythm in striatal dopamine receptors.

It is thus not surprising that seasonal and/or diurnal variation was revealed both in movement disorders and in addiction. For example, there exists an interconnection between diurnal rhythms and Parkinson’s disease [102]. Similarly, there is an interconnection between addiction and circadian rhythms [103].

### 3.4. Contribution of Signaling Interactions to the Regulation of Behavioral Outcomes

In previous subsections, we have mentioned the contribution of dopamine and acetylcholine signaling systems to locomotor activity, addiction, and biological rhythm. Here we will mention specific interactions of these systems with respect to behavioral outcomes.

The dopaminergic transmission is, for example, increased in mice lacking M_1_ muscarinic receptors [63], these mice reveal hyperactivity and have elevated response to amphetamine. This paper thus clearly demonstrates that disturbed equilibrium between two neurotransmitter systems (by M_1_ muscarinic receptor disruption) leads to behavioral changes, as well as to a distinct reaction to an addictive drug—amphetamine. Similarly, in M_4_ muscarinic receptor knockout mice, there was an increase in basal locomotor activity and greatly enhanced locomotor responses following drug-induced activation of D_1_ dopamine receptors [20]. M_4_ muscarinic receptors were also shown to modulate dopamine-dependent behavior [22] such as hyperlocomotor responses induced by a dopamine agonist, amphetamine, or cocaine, the reduction of cataleptic effects of antipsychotic drugs, or the amphetamine-induced behavioral sensitization. Moreover, dopamine eflux was enhanced in nucleus accumbens in these mice. The behavioral effects of cocaine were also inhibited by an allosteric enhancer of M_4_ muscarinic receptors [76]. M_1_ and M_4_ muscarinic receptor blockade reduces D_1_R agonist-induced dyskinesia in rat [21]. M_4_ muscarinic receptor activity regulation of dopamine release and signaling, the potential sources of acetylcholine that can regulate M_4_ muscarinic receptor activity, and the implications of targeting M_4_ muscarinic receptor activity for the treatment of the motor symptoms in movement disorders have been reviewed [38]. In mice with M_5_ muscarinic receptor lacking, a muscarinic agonist was less potent in increasing potassium-stimulated dopamine release in striatal slices which could correspond to decreased self-administration of low to moderate doses of cocaine [24]. In mice totally lacking forebrain acetylcholine there was an enhanced frequency-dependent striatal dopamine release and increased exploratory motor behavior (in a novel environment). Involvement of dopamine in the exploratory motor phenotypes observed in these mutants is indicated by their altered sensitivity to the dopamine D_2_ receptor antagonist [68]. The neurotransmitter levels are associated with Parkinson’s disease severity in rat models [48]. Acetylcholine also encodes presynaptic plasticity at glutamatergic synapses after amphetamine exposure [77] which was affected by dopamine D_1_ receptors and it was correlated with locomotor responses after drug challenge. Functional interaction between α_6_β_2_-containing nicotinic and adenosine A_2A_ receptors in striatal dopaminergic terminals affected locomotor sensitization, which could have therapeutic consequences for smoking, Parkinson’s disease and other dopaminergic disorders [31]. Robust motor impairment elicited by α_4_β_2_ nicotinic antagonist is characterized by hypolocomotion, akinesia, catalepsy, clasping, and tremor in mice with a point mutation in α_4_ nicotinic receptor subunit [33]. In these mice, acetylcholine evoked dopamine release from striatal synaptosomes with agonist hypersensitivity. On the other hand, there are also findings [78] that biologically relevant concentrations of cocaine inhibit nicotinic-mediated currents that alter dopamine release in the dorsal and ventral striatum. Partial inhibition of nicotinic receptors by cocaine reduces dopamine release. This cocaine-induced shift favoring phasic dopamine release may contribute to the enhanced saliency and motivational value of memories and behaviors [78]. Amphetamine-induced stereotypy (consisting of confined sniffing and licking behaviors) in mice overexpressing the vesicular acetylcholine transporter, which consequently increases acetylcholine release and changes dopamine release, was greatly increased [70]. The behavioral characteristics of nicotine addiction, together with the effects of nicotine, on the function of mesolimbic and mesocortical dopamine projections in the mesocorticolimbic circuit were also reviewed [79]. Striatal cholinergic—dopamine interactions in behavior were also reviewed [41].

## 4. Hierarchical Model of the Interaction between Dopamine and Acetylcholine Signaling Systems

There is a tight interaction between dopamine and acetylcholine signaling in the striatum. Dopaminergic neurons express M_3_ and M_5_ muscarinic receptors and nicotinic receptors containing the α_4_, α_6_, and β_2_ subunits. Conversely, cholinergic interneurons express the dopamine receptors D_1_ and D_5_. In addition, dopaminergic neurons are autoinhibited by D_2_ dopamine receptors, and cholinergic neurons are autoinhibited by M_2_ and M_4_ muscarinic receptors [3,20,28,33,35,56,64,90]. These autoreceptors, in general, inhibit the release of specific neurotransmitters, i.e., of dopamine or acetylcholine, respectively. All neurons in the striatum reveal rhythmic changes in membrane potential; thus, they can be considered pacemakers [27]. Functional interactions exist at the dopamine and acetylcholine levels, as can be deduced from the existence of dopamine/acetylcholine receptors affecting acetylcholine/dopamine release from cholinergic/dopaminergic neurons. For example, an increase in dopamine release is activated by stopping acetylcholine release [10]. Rhythmic firing of cholinergic interneurons and loss of autoinhibition of acetylcholine release by M_4_ muscarinic receptors result in the unregulated release of acetylcholine. Thus, some authors concluded that the coordinated timing or synchrony of the direct and indirect pathways is critical for refined movements [35]. In addition, changes in neurotransmitter ratios (i.e., dopamine/acetylcholine) [10] are considered prominent factors in striatal contributions to Parkinson’s disease [11] and Huntington’s disease. In general, drugs (stimulants, with the exception of cannabis and opiates) increase striatal dopamine release, which in turn decreases the numbers of dopamine receptors (D_2_ and D_3_) [17]. Some drugs can potentiate the electrically evoked release of both dopamine and acetylcholine [73]. Both dopamine and acetylcholine reveal diurnal variations. Dopamine has a peak in the daily rhythm in the dark (active) period, and this rhythm is circadian [87]. D_2_-like dopamine receptors reveal ultradian rhythms with two peaks of maximal density in the light (i.e., in the non-active) period [89]. Similar to dopamine, acetylcholine also reveals diurnal variations with a peak at 24:00 (i.e., in the dark/active period) [93]. Muscarinic receptors also reveal circadian variation [98,99] with two troughs, one in the light period and one in the dark period. Recently, it was observed that reward is modulated by the circadian system [91], and some drugs can trigger reprogramming in circadian gene expression [52]. Similarly, behavioral changes (significant hyperactivity and hypoactivity during the light and dark phases, respectively) were present in an animal model of the preclinical stage of Parkinson’s disease [92]. The striatum is one of the key structures responsible for increased locomotion in the active (dark) period in mice lacking M_4_ muscarinic receptors [100], and this brain region was the only structure in which M_1_ muscarinic receptors played a role in locomotor biological rhythm regulation; in others (i.e., in the thalamus and intergeniculate leaflet), only M_4_ muscarinic receptors were responsible for this regulation.

**Figure 2:**
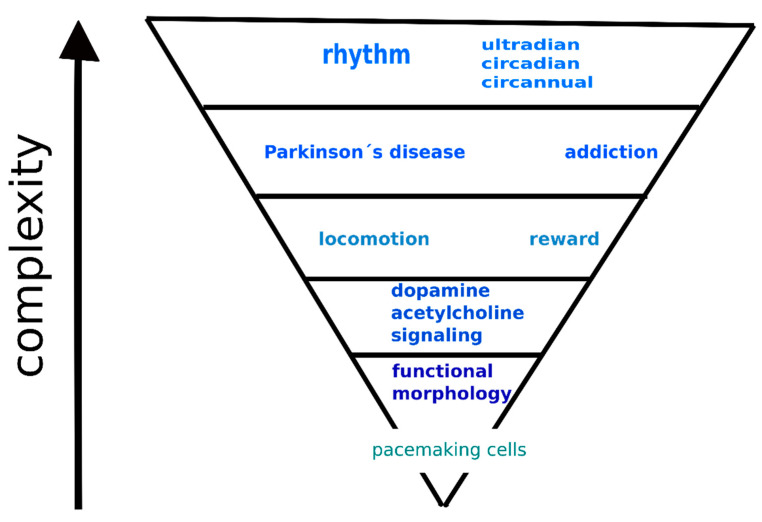
Hierarchical model of striatal interactions between the dopamine and acetylcholine neurotransmitter systems.

Thus, it is possible to propose a hierarchical model of the interactions between the dopamine and acetylcholine signaling systems in the striatum (see Figure 2). The complexity of interactions between these two neurotransmitter systems gradually increases, as further described below. The base of this model represents functional morphology, with respect to the fact that all neurons in the striatum can operate as pacemakers. The next highest mode of interaction between these two neurotransmitter systems is interaction at the neurotransmitter/receptor/signaling level. Furthermore, these interactions contribute to locomotor activity regulation and reward behavior. Relatively recently, some new findings revealed that both functions exhibit biological rhythmicity. These rhythms can be ultradian, circadian, or circannual or can show all rhythmic components. Similarly, pathologies such as Parkinson’s disease and addiction also exhibit rhythmic changes. For examples of how these interactions affect behavior, see Section 3.4.

## 5. Conclusions

We propose here a hierarchical model of the interaction between dopamine and acetylcholine signaling systems in the striatum. The lowest level in this model represents functional morphology and comprises connections between medium spiny neurons and cholinergic neurons with respect to the fact that all neurons in the striatum can operate as pacemakers. The next highest mode of interaction between these two neurotransmitter systems is interaction at the signaling level, i.e., on the level of neurotransmitter concentration, receptor number, and specific signaling cascades. Furthermore, these interactions contribute to locomotor activity regulation and reward behavior. The pathological states of Parkinson’s disease and addiction represent another level of interaction between these two neurotransmitter systems. Also, specific changes can also be described in the dopamine and acetylcholine signaling, starting from neurotransmitter concentrations up to signaling cascades modifications. Relatively recently, some new findings on diurnal variation in striatal dopamine/acetylcholine functions have been revealed. Thus, it is possible to view locomotor activity and reward behavior as dynamic functions that do not have a stable degree of intensity but are subject to change. Both functions reveal biological rhythmicity and can be ultradian, circadian, or circannual, or can include all rhythmic components. Similarly, pathologies such as Parkinson’s disease and addiction also reveal rhythmic changes.

## Figures and Tables

**Table 1:** Main cell types in the striatum ^1^.

Neuron Type	Principal Neurotransmitter	Main Dopamine Receptor Subtype Expressed	Main Pathway/Efferent Structure (or Neurons) Activated
Medium spiny neurons (type I)	GABA	D_2_	Indirect pathway/GP
Medium spiny neurons (type IIa)	GABA	D_1_	Direct pathway/GP, EP, SN
Medium spiny neurons (type IIb)	GABA	D_1_	Direct pathway/GP, SN
Interneurons	Acetylcholine	D_1_, D_5_	Medium spiny neurons

^1^ According to [3,20,28]. Medium spiny neurons are also activated by the thalamostriatal pathway via the excitatory amino acids glutamate and aspartate. GP: globus pallidus, SN: substantia nigra, EP: entopeduncular nucleus.
